# A critical interpretive synthesis of the constructed identities and experiences of refugees, asylum seekers, and undocumented migrants in relation to accessing primary care services in the UK

**DOI:** 10.1016/j.jmh.2025.100389

**Published:** 2025-12-25

**Authors:** Jeniffer Jeyason, Georgia B. Black

**Affiliations:** Queen Mary University of London, United Kingdom

**Keywords:** Primary care, Critical interpretive synthesis, Refugees, Asylum seekers, Immigrants, Migrant health, Healthcare access, Post-colonial

## Abstract

Refugees, asylum seekers, and undocumented migrants face significant structural and social barriers when accessing, or attempting to access, primary care delivered by the NHS in the United Kingdom. The aim of this research is to conduct a critical interpretive synthesis of primary research articles, identified via a systematic search of several online databases. This review approach was chosen in order to develop a new synthesis of the constructed identities and experiences of refugees, asylum seekers and undocumented migrants, and contribute to a further understanding of the barriers and facilitators that these populations face in relation to primary care access. This research used an interdisciplinary framework, guided by Penchansky and Thomas’ theory of healthcare access, as well as Bhaba’s, Spivak’s and Berry’s post-colonial theories. Differences in social cues, the impact of the past, and the role of communities are examples of the various barriers and enablers that refugees, asylum seekers, and undocumented migrants face in relation to primary care in the UK. This analysis generated a new model, the *Tangibility of Access*, which theorises that recognising the tangibility of various identities and experiences can provide a deeper insight into persisting barriers of primary care access. These findings highlight the implications of assuming the nature of identities and experiences in research, and identifying how differences in the knowledge of these vulnerable groups between research and policymaking can lead to continued difficulties surrounding primary care access. Further interdisciplinary research is necessary to determine the causal effects of deterrents to approaching primary care, and provide insights into how the quality of primary care can be improved, especially concerning intangible identities and experiences. A holistic perspective is needed to challenge assumptions regarding the identities and experiences of refugees, asylum seekers, and undocumented migrants, which may prove harmful to help-seeking behaviour if not confronted.

## Highlights

• Forced immigrants face difficulties in recognising and responding to help-seeking cues.
• This article conducts a critical interpretive synthesis of research on primary care access in the UK.
• From the analysis, we generated the *Tangibility of Access* theory.
• Penchansky and Thomas’ theory and post-colonial theories enabled a critical discussion of barriers and facilitators to access.
• Interdisciplinary research is necessary to understand how identities and experiences of vulnerable groups are conceptualised.

## 1 Introduction

Migration is characterised by the physical, mental, socioeconomic, and political impact on the individuals who partake in it as a result of circumstances outside of their control. The United Nations High Commissioner for Refugees (UNHCR) defends the global right to healthcare access for migrants, as highlighted in the Global Compact on Refugees and the 2030 Agenda for Sustainable Development (UNHCR, 2021), especially with relation to improving continuity of care (Harris et al., 2022). The newly launched ‘World report on the health of refugees and migrants’, published by the World Health Organisation (2022), highlights the urgent need to identify how healthcare systems can effectively respond to challenges faced by patients belonging to these vulnerable groups. In the UK, most patient contacts with the NHS are in primary care (Newell, 2016). Primary care constitutes of NHS services, such as general practices and dental and optometry support, as well as community centres external to NHS hospital trusts (Care Quality Commission, 2018). This paper is focused on primary care delivered by the NHS due to its national accessibility, and existing frameworks surrounding specific care of asylum seekers, refugees, and undocumented migrants (Office for Health Improvement and Disparities, 2014).

Despite national availability, barriers to and within primary care can reduce the effectiveness of consultations for refugees, asylum seekers and undocumented migrants, and even deter them from approaching these services altogether (Giuntella et al., 2018). For example, studies have consistently identified linguistic barriers to care (Bhatia and Wallace, 2007; Bellamy et al., 2015; Jallow et al., 2021; Tankwanchi et al., 2021), despite responsibility for practitioners in primary care to provide access to translation services (NHS England Primary Care Commissioning, 2018). Lack of clarity surrounding migrant status in research concerning primary care has also been observed, with unclear distinctions between refugees and asylum seekers (Masocha and Simpson, 2012), largely due to poor data collection by healthcare systems (Rechel et al., 2012). Barriers to primary care for refugees, asylum seekers, and undocumented migrants have led to increased reliance on Accident and Emergency (A&E) services (Graetz et al., 2017; Phung et al., 2020; Aspinall, 2014). Since 2010, A&E services in the UK have faced increased pressure due to unsustainable demand, especially during winter (Secretary of State for Health, 2017). In this context, refugees, asylum seekers, and undocumented migrants may have distressing healthcare experiences, particularly in circumstances where they were unaware that more appropriate services were accessible.

Notably, primary research on the experiences of refugees, asylum seekers, and undocumented migrants with primary care services have largely come from the perspective of medicine itself (Lindenmeyer et al., 2016; Nellums et al., 2018). Little interdisciplinary research has been conducted to analyse and interpret the experiences of asylums seekers, refugees, and undocumented migrants, which may offer a greater insight into barriers of healthcare access (Nellums et al., 2018). A critical assessment of the current evidence surrounding UK primary care in relation to social, cultural, and historical ties may give a greater insight into how policies and recent interventions can improve the relationship between these vulnerable groups and the healthcare touchpoint.

This paper presents a systematic review and critical interpretive synthesis of evidence relation to the identities and experiences of refugees, asylum seekers, and undocumented migrants, in relation to primary care services in the UK, and how they are conceptualised in research as barriers and facilitators. The following research questions guide this research:

1. How have the identities and experiences of refugees, asylum seekers, and undocumented migrants been conceptualised as barriers and facilitators of primary care access?
2. How might an interdisciplinary perspective improve the assessment of primary care access?
3. What are the research and policy implications regarding the accessibility of primary care for refugees, asylum seekers and undocumented migrants?

## 2 Materials and methods

We use definitions provided by The British Medical Association (2019), based on the 1951 UN Convention. These definitions are the following:

- Refugees are populations who have fled their home country, due to circumstances out of their control, which poses a risk of persecution and, or, a risk to life.
- Asylum seekers are those who have fled under similar circumstances as refugees but have appealed to the host country to be recognised as a refugee, and are therefore given no confirmation regarding their ability to remain.

Furthermore, there is significant concern for undocumented migrants who are not recognised under law, but are just as in need for access to healthcare support. Therefore, this group will also be included in this research.

### 2.1 Ethical considerations

Regarding our positioning as authors, GB is a qualitative health services researcher and JJ is an interdisciplinary student, both with particular interest in primary healthcare access. Vulnerable groups are the subject of this research which requires ethical consideration of the interpretations which are made by authors who have always lived and worked in the UK with no lived experience of seeking refuge or asylum. Ethical consideration is also warranted regarding the dependency on reliable translations, which may not capture all linguistic nuances.

### 2.2 Critical interpretive synthesis

This research used a critical interpretive synthesis approach, rooted in meta-ethnography (Dixon-Woods et al., 2006). This methodology was selected as it encompasses the strength of a systematic review in its search strategy, whilst stimulating critique and generating theory to develop new insights (Dixon-Woods et al., 2006). Penchansky and Thomas’ (1981) theory of healthcare access and post-colonial theory (Bhaba, 1994; Spivak, 1995; Berry, 1997) were used to guide the interpretation and synthesis of the literature. Barriers and facilitators to access were derived inductively from synthesis of the included studies and mapped against the six domains of the Tangibility of Access Model.

### 2.3 Theoretical framework for the review

The definition of access can vary throughout the literature, thus, required definition. We based our theoretical framework on Penchansky and Thomas’ (1981), who deconstruct healthcare access into the dimensions of Availability, Accessibility, Accommodation, Affordability and Acceptability, to measure the efficacy of healthcare services. The use of Penchansky and Thomas’ theory to assess healthcare access is well-supported in the literature (Fradgley et al., 2015; Retrouvey et al., 2019; Otieno et al., 2020; Oginni et al., 2021). For example, it was effectively used in an interview-led primary research study concerning primary care access for asylum seekers and refugees (Kang et al., 2019). Papers were organised according to how it relates to the relevant categories under Penchansky and Thomas’ (1981) theory, and these categories themselves were subject to critique.

Availability is the inherent capacity of physical and human resources within primary care services to help the patients that require them (Penchansky and Thomas, 1981). Examples include language interpretation services, consultations, and the practitioners required to run them. Accessibility is the physical ability of refugees, asylum seekers, and undocumented migrants to reach healthcare services (Penchansky and Thomas, 1981). Accommodation encapsulates the ability of primary care facilities to meet the needs of vulnerable groups through their formal systems, such as booking appointments, registering at a practice, or the grounds for walking in (Penchansky and Thomas, 1981). Affordability refers to the costs of engaging with primary care services (Penchansky and Thomas, 1981), and how this knowledge, or lack of knowledge, might affect help-seeking behaviour. Acceptability pertains to whether the beliefs that refugees, asylum seekers, and undocumented migrants hold about primary care services, and the practitioners running these services, are in line with the intended goals of primary care (Penchansky and Thomas, 1981). It also encompasses the attitudes and beliefs of practitioners towards refugees, asylum seekers, and undocumented migrants. Another strand introduced by Saurman (2016), as an addition to the Penchansky and Thomas’ framework, is Awareness. This is regarding the access to information about the primary care services available to vulnerable groups. Due to this being an important aspect of primary care access for refugees, asylum seekers, and undocumented migrants, it was incorporated in this research, as successfully illustrated by Kang et al. (2019).

### 2.4 Post-colonial theory

Post-colonial theory studies the impact of social, political, historical, and cultural ties on healthcare access (Racine, 2009; Zaman et al., 2017; Berg et al., 2019). The identities of refugees, asylum seekers, and undocumented migrants harbour cultural and social complexity that is in constant flux between multiple pressure points, such as familial duties and occupational expectations (Hack-Polay et al., 2021). The term ‘post’ does not carry temporal significance, but rather attempts to address how colonialism is still present within societies via its after-effects (Said, 1978). We drew on the post-colonial works of Homi Bhaba (1994), Gayatri Spivak (1995), and John Berry (1997) in conceptualising our review.

Bhaba (1994) introduces the concept of hybrid identities and spaces. This theory provides a critical outlook of ‘singularities’ and ‘binaries’, as these terms do not acknowledge changing cultural elements that shape the identities and experiences of individuals. Bhaba argues for ‘cultural translation’ which is a critique of the social and cultural expectations for migrants to conform to a new society in a passive, unilateral manner.

Spivak (1995) presents the concept of the subaltern, derived from the military use of the term, to refer to individuals who are unable to deliver opinions first-hand, largely due to socioeconomic and political circumstances. This sheds light on the positions of researchers and mediators who document the experiences of refugees, asylum seekers, and undocumented migrants, and may speak on their behalf. This concept enabled a critical discussion of methods used in research, and possible implications.

Berry’s (1997) model of acculturation argues that the integration of host and migrant populations provides a beneficial population dynamic for inhabitants. Separation, Integration, Marginalisation or Assimilation are the four categories within Berry’s model, and have been used in UK migration policies (Ndofor-Tah et al., 2019). There is an implied positive value of integration and the implied negative value of marginalisation in this theory. Inclusion of this model offered insight into possible assumptions that are being made about the expectations of refugees, asylum seekers, and undocumented migrants within the host community, and whether this is present in research.

To situate our Critical Interpretive Synthesis within contemporary theoretical debates, we draw explicitly on two converging strands in the health-inequalities literature. First, the concept of structural vulnerability, which seeks to operationalise how social, legal, and economic hierarchies become embodied and shape clinical risk, offers practical entry points for connecting power-centred interpretation with clinical and service responses (Bourgois et al., 2017; Thompson-Lastad et al., 2017). Second, intersectionality debates in population health raise methodological and translational questions about how intersecting social positions (e.g. legal status, race/ethnicity, gender, class) are to be theorised, measured, and acted upon in policy and practice. Recent methodological work has advanced analytic intercategorical methods and emphasised the importance of using intersectionality both as an emancipatory research stance and as an operational tool in quantitative and qualitative studies (Bauer, 2014; Agénor, 2020; Bauer and Scheim, 2019). These debates explain our interpretive choice to foreground relational power and lived experience in the Tangibility of Access model, while also signalling avenues for future mixed-method or evaluative research to test and operationalise our theoretical claims.

### 2.5 Search strategy

Due to the interdisciplinary nature of this research, papers were screened from databases of different subject areas. Considering the time limit of the research, without sacrificing coverage, this study selected Ovid Medline, APA PsycINFO, Web of Science, IBSS, ASSIA, and GEOBASE as appropriate databases to consult.

A preliminary search of databases was conducted to identify appropriate search terms. A full list of terms associated with primary care and terms for refugees, asylum seekers, and undocumented migrants were combined with the Boolean operator ‘and’, as indicated in Appendix A. Papers were filtered according to the inclusion criteria by title and abstract, followed by a full-text reading. We then conducted a quality assessment of these papers before analysis.

### 2.6 Inclusion & exclusion criteria

The first strand of the inclusion criteria includes substituted terms for refugees, asylum seekers, and undocumented migrants, and the second strand relates to primary care. To reduce sampling bias, the search combined index terms and manual search terms. The 2012 Health and Social Care Act, which primarily applies to England, was used as a policy benchmark to delimit the ten-year search window, reflecting major reforms to NHS primary-care commissioning. However, studies from all UK nations were included where their findings related to NHS-funded primary-care systems operating under comparable principles.

Wider inclusion criteria include papers:

- Based in, or including research on, the UK
- Published within the last 10 years to consider the effect of the Health and Social Care Act (2012)
- Written in English

Wider exclusion criteria include papers focused on:

- Secondary care or beyond
- Community-led healthcare external to the NHS which may have a different system pertaining access and is often location-dependent
- Emigration of a population
- Economically-motivated migration, or other forms that are not under forced migration

## 3 Results

### 3.1 Search outcome and quality assessment

Database searches were conducted from 1st January 2022 – 31st March 2022. From the initial search, 9286 papers were identified. It was deemed necessary for the search to be refined further in the Web of Science database as it initially generated 25,376 papers. The best selected course of action was to create an additional query consisting of terms synonymous to the United Kingdom (see Appendix A). The PRISMA chart (Page et al., 2021), maps our screening process (see Appendix B).

Following searching, 8478 study titles and abstracts were screened by the first author after removing duplicates, dissertations, theses, and protocols, with uncertainties resolved through discussion with the second author. Due to time constraints, papers were rejected if there was a lack of reference to the type of immigrant status in the title or abstract, distilling the search to 367 papers. The first author completed full-text review of these studies with a sub-sample reviewed by the second author, and re-assessed against the inclusion criteria. Disagreements were resolved through discussion. There were no outstanding disagreements following this process. There were 49 papers remaining following full-text review. Quality appraisal followed the NHS National Electronic Library for Health framework, adapted from Dixon-Woods et al. (2006), evaluating methodological transparency, sampling, and analytical coherence. A summary of appraisal outcomes is presented in Appendix C. No papers were excluded as they all satisfactorily met the criteria.

Of the 49 included studies, 30 were qualitative (interviews and focus groups), 9 used mixed methods, and 10 were quantitative surveys. 7 were multi-region or UK-wide, while 42 focused on specific regions including London, Northern England, Scotland, Wales, and Northern Ireland.

### 3.2 Critical interpretive synthesis: an understanding of access to primary care for refugees, asylum seekers, and undocumented migrants with post-colonial insights

Our synthesis of the literature began by using Penchansky and Thomas’ framework to chart the extracted data. As we critically reviewed findings by drawing on post-colonial perspectives, we found that Penchansky and Thomas’ framework was limited in its ability to capture our analysis due to the interdisciplinary nature of our interpretations. Thus, we developed a model outlining the *Tangibility of Access* which builds on the five elements of Penchansky and Thomas’ (1981) theory, in addition to Saurman’s (2016) domain of Awareness.

### 3.3 *Tangibility* of access model

**Fig. 1:**
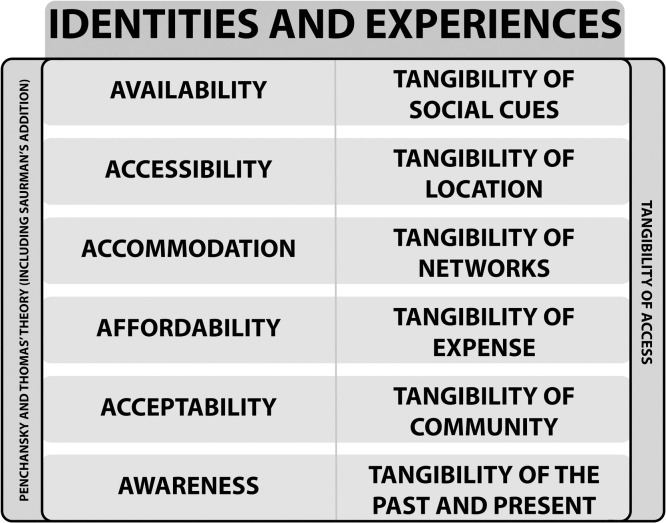
Illustration of the *Tangibility of Access* model in relation to the identity and experiences of refugees, asylum seekers and undocumented migrants.

In this paper, a ‘tangible state’ is defined as experiences and identities presented by refugees, asylum seekers, and undocumented migrants that possess physical representation, in the form of objects or documentation, such as policies, records, and reports. An ‘intangible state’ refers to identities or experiences without a physical manifestation but that shape access - for example, trust, fear, stigma, or emotional responses arising from previous encounters with healthcare. The model consists of six domains in relation to access: social cues, location, networks, expense, community, and the past and present (Fig. 1). We consider how tangible and intangible identities and experiences in each domain affect primary care access for refugees, asylum seekers, and undocumented migrants. We have differentiated barriers relating to identity from those relating to experience, where ‘identity’ refers to attributes legally or socially ascribed (e.g. refugee status, gender), whereas ‘experience’ captures situational interactions such as discrimination or support within care encounters. Each element is illustrated below.

#### 3.3.1 Availability; tangibility of social cues

**Fig. 2:**
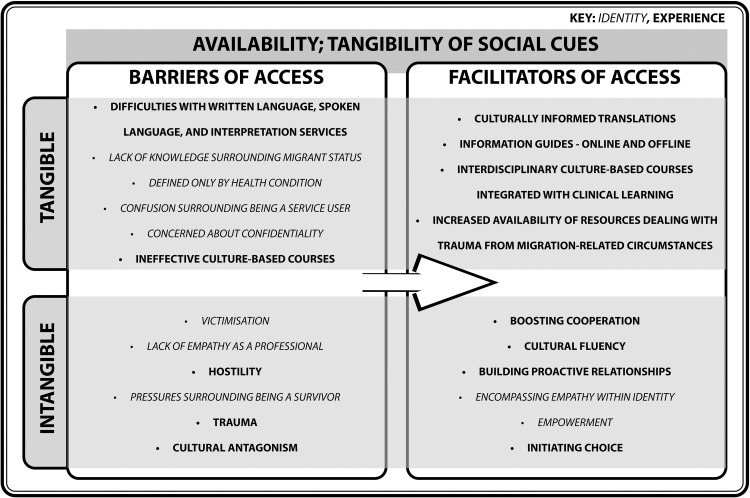
Turning barriers into facilitators of access within the domain of Availability; Tangibility of Social Cues.

Available resources provided by primary care depend on social cues, such as language interpretation and cultural fluency, for successful interaction by refugees, asylum seekers, and undocumented migrants (Fig. 2). Barriers listed in the left column do not correspond to the thematically linked facilitators in the adjacent row. This section reveals how negative social cues such as hostility, antagonism, trauma, and victimisation can hinder access to care.

It is difficult to measure the efficacy of primary care resources by only looking at what resources are available in a given space and time. Many studies cite a lack of interpreters or language problems during consultations (Alkahtani et al., 2014; Armitage et al., 2021; Cheung and Phillimore, 2017; Falla et al., 2017; Gazard et al., 2015; Goodwin et al., 2021; Higginbottom et al., 2014; Hoare et al., 2020; Hollis, 2019; Isaacs et al., 2020; Kaneoka and Spence, 2020; Kang et al., 2019; Kearns et al., 2017; Morrice, 2017; Murphy et al., 2020; Nellums et al., 2021; Paudyal et al., 2021; Perry et al., 2020; Piacentini et al., 2019; Priebe et al., 2013; Rabiee and Smith, 2013; Rayment-Jones et al., 2021b; Samkange-Zeeb et al., 2020; Scott et al., 2022). Most of the aforementioned papers suggest that an improvement in the reliability of translation-aided resources is required to facilitate healthcare access. This form of recommendation opposes the negative connotation in Berry’s (1997) model which suggests that maintaining cultural ties, but unsuccessfully adapting to the host culture, by not learning the language in this case, is deemed as ‘separation’. Although socioeconomic policies are guided by the concept of integration (Ndofor-Tah et al., 2019), which is a balance between cultural roots of the host country and the country they migrated from, recommendations from research emphasise a greater responsibility for the host community to provide relevant resources. Additionally, fluency in a language does not necessarily equate to cultural fluency. Multiple countries may share the same language, but existing colloquial differences signify how culture can mould the usage of certain expressions. When considering how to make online resources available (Quinn, 2014; Poduval et al., 2015; Kaneoka and Spence, 2020; Samkange-Zeeb et al., 2020), it may be effective to provide informational guides to medical terminologies, and engaging a culturally informed translation service for these groups. A proposed recommendation is to empower patients to decide who they trust to interpret their concerns (Rayment-Jones, 2021b). This highlights a move towards a proactive relationship between primary care and patients where refugees, asylum seekers, and undocumented migrants can feed into appropriate medical care, whilst ensuring confidentiality and accuracy.

Tangible and intangible labels attached to identities can affect the understanding of what resources should be available for refugees, asylum seekers and undocumented migrants who have traumatic experiences. Refugees report more health problems compared to British citizens (Alkahtani et al., 2014), with some refugees, asylum seekers, and undocumented migrants harbouring mental and physical abuse from war (Negron, 2018). This suggests the increased need for resources that specifically deal with trauma. However, there are multiple identities attributed to these vulnerable groups, such as ‘service users’, their migrant status, ‘sufferers’, and ‘torture survivors’ (Negron, 2018). Each of these labels have their own tangibility; the first two labels are legal definitions which correspond to a visible position within the healthcare system. The last two labels are intangible as it relates to a negative mental impact, and encompasses one’s identity as a victim. This demonstrates how different labels carry various connotations. There is also the concern that patients do not want to be defined by their conditions. For example, a participant in a qualitative study of HIV-positive asylum seekers in Scotland (qualitative interview study, *n* = 25) stated, “I don’t want to be reminded all the time about my status; HIV is not my second name” (Palattiyil and Sidhva, 2021, p. 271), illustrating the effect that constructed identities can have for these populations. The tangibility of identities may carry different social cues for these groups, and reflects how the usage of one label might influence a narrative.

Interactions with available resources are also affected by intangible social cues, such as cultural values and empathy, which may promote effective healthcare consultations. Several papers uncover concerns surrounding trust, confidentiality, a lack of cooperation, or direct interference from interpreters (Armitage et al., 2021; Burchill and Pevalin, 2014; Kang et al., 2019; Rayment-Jones et al., 2021b). Providing culture-based resources for primary care practitioners has been recommended (MacVicar et al., 2015), and implemented in some practices (Hussain et al., 2021). Training courses are often delivered by external organisations with no direct familiarity with primary care, alongside a lack of involvement from vulnerable groups (Hussain et al., 2021). In this context, it implies that these populations are the subaltern, in line with Spivak’s (1995) theory, as they are not able to provide feedback on training concerning their care. Constructing the identities of refugees, asylum seekers, and undocumented migrants in educational resources without holistic input may lose empathetic ties between practitioners and patients.

#### 3.3.2 Accessibility; tangibility of location

**Fig. 3:**
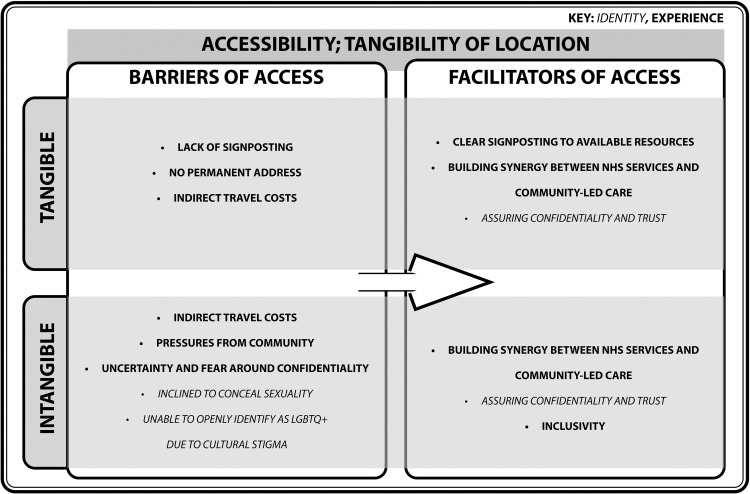
Turning barriers into facilitators of access within the domain of accessibility; tangibility of location.

Penchansky and Thomas’ (1981) theory refers to the physical access to services. However, incorporating intangible identities and experiences, such as sexuality and mental health issues, uncover further barriers that these vulnerable populations face (Fig. 3).

Despite services being physically available, there are tangible barriers including financial problems, lack of tailored signposts to primary care access, and issues with permanent addresses. In one study, 9 out of 50 parents, who identified as refugees, faced financial difficulties when looking to visit a General Practice, compared to no issues mentioned by the control group (Alkahtani et al., 2014). This is especially difficult for those who do not have a permanent address (Mudyarabikwa et al., 2022), as it inhibits continuity of care which would disrupt a development of trust with a practitioner. Coupling access to NHS services with community-led care has proven effective in improving accessibility, especially as participants expressed that these services were within walking distance (Jackson-Blott et al., 2015), enabling reduced travel costs.

Intangible barriers with respect to travel and location consist of whether, or, how refugees, asylum seekers, and undocumented migrants align themselves with the host population. Berry’s (1997) model suggests that the process of separation between an individual and the host culture occurs due to an increased likelihood for refugees, asylum seekers, and undocumented migrants to be pulled to communities that share their culture. People who have forcibly migrated due to a lack of rights for the LGBTQ+ community may find prevalent homophobic attitudes carried within their cultural community in the host country (Mudyarabikwa et al., 2022). Even if there are available support services nearby, it may not be accessible due to the fear of community members being aware of the patient’s visit, and fear of a confidentiality breach. Therefore, physical accessibility cannot solely be used as a measure of whether refugees, asylum seekers, and undocumented migrants have access to the service, as this journey is shaped by the intangible perceptions of its consequences. This brings to light the importance of increasing dialogue around inclusivity and confidentiality.

#### 3.3.3 Accommodation; tangibility of networks

**Fig. 4:**
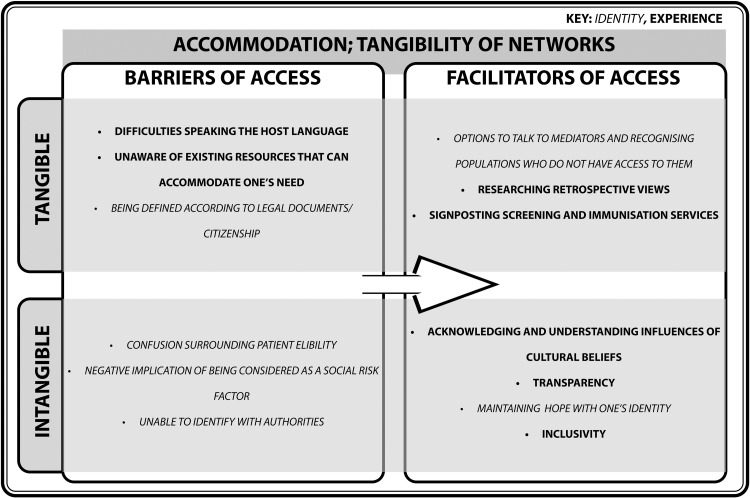
Turning barriers into facilitators of access within the domain of accommodation; tangibility of networks.

Knowledge of how services can appropriately accommodate needs, such as via appointment bookings or walk-in systems, can assist help-seeking behaviour. Social networks and healthcare networks can influence how refugees, asylum seekers, and undocumented migrants recognise their eligibility to care (Fig. 4).

In connection with the prior two domains, tangible and intangible linguistic barriers, social cues, and locations are key barriers of tapping into healthcare-based accommodation. One way that these populations can gain knowledge about nearby healthcare services is by accessing information via social networks, such as charities. One study recruited participants via Refugee Action, a charity which supports this vulnerable group (Alkahtani et al., 2014). This paper found that all refugees were registered with a GP, a majority booked and attended appointments regularly, and all refugee children except one were immunised (Alkahtani et al., 2014). However, not all refugees, asylum seekers, and undocumented migrants have these networks. Studies that use mediators to recruit participants are therefore not representative of the identities and experiences of populations who do not have contact with a mediator (Kang et al., 2019). This may advance the assumption that most refugees understand primary care accommodation. However, contact with the charity may have contributed to signposting participants to these services beforehand (Alkahtani et al., 2014). Research surrounding vulnerable groups are subject to such sampling biases due to difficulties of accessing these populations via other means (Kaneoka and Spence, 2020; Palattiyil and Sidhva, 2021; Paudyal et al., 2021; Scott et al., 2022). A study on an oral health intervention program adopted ‘snowball sampling’, which harnesses the social networks of initial participants to facilitate recruitment (Yuan, 2019). Invitation by a known contact can encourage trust and build motivation to engage with the study. However, it is likely that this network shares cultural beliefs, thus, it is difficult to extrapolate findings to populations who may not possess similar views. There are potential underlying assumptions supported by these studies which may affect the conceptualisation of what vulnerable populations experience. This signifies the importance of integrating diverse and retrospective views of those who have navigated healthcare services as a refugee, asylum seeker, and undocumented migrant to improve recommendations to improved accommodation.

To ensure that primary care is accessible, tangible identities, including the eligibility to care, and intangible needs, such as confidence, certainty, empathy, should both be accommodated. There are reports of incorrectly assessing the eligibility of patients to primary care services, or, requesting documents that are not legally required to be seen (Papageorgiou et al., 2020; Feldman, 2021). This can cause a stressful and unwelcoming environment where the identities of help-seeking individuals are reduced to physical documentation of which the criteria is poorly defined. Interventions that integrate transparency, cultural attitudes, and support improved participation (Bahu, 2019; Jackson-Blott et al., 2015; Yuan, 2019). However, using binaries to construct identities can reduce workshop engagement if participants feel that they are misunderstood, which can, in turn, disrupt help-seeking behaviour. Bhaba (1994) criticises binaries as a reductive construction of identity, such as only focusing on migrant status or ethnicity. For instance, a study about the improvement of maternal care constructed asylum seeker or refugee status as a ‘social risk factor’, alongside issues such as drug abuse, or non-English speaking (Rayment-Jones et al., 2021b). Whilst this approach is vital to identify how this identity may affect maternal care, it harbours an intangible negative connotation, whereas this might be a source of hope or strength for those who have arrived at a new community in pursuit of safety. Uncomfortable experiences with authorities tend to dissolve such feelings and lead to negative associations with this identity (Quinn, 2014; Hoare et al., 2020), illustrating how experiences can shape how patients perceive their identities.

#### 3.3.4 Affordability; tangibility of expense

**Fig. 5:**
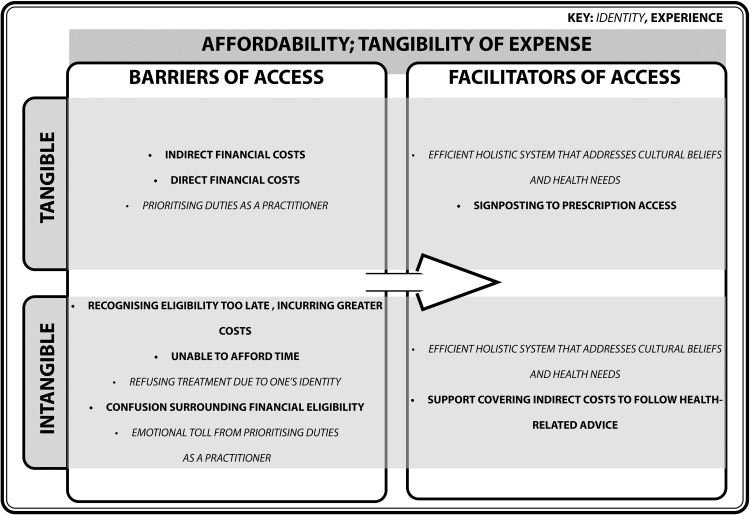
Turning barriers into facilitators of access within the domain of Affordability; Tangibility of Expense.

The perception of affordability should encompass both indirect and direct financial tolls, as well as the cost of time to attend primary care services (Fig. 5).

The domain of Affordability feeds into Awareness and Accommodation, as there are issues regarding confusion of financial entitlements to primary care accommodation, and indirect monetary expenses. This increases the likelihood of requiring secondary care, which causes greater financial and strain for these services (Murphy et al., 2020; Papageorgiou et al., 2020). Whilst direct costs of registering with primary care are covered by the NHS for these vulnerable populations, some are unaware of their entitlements (Murphy et al., 2020; Nellums et al., 2021; Mudyarabikwa et al., 2022), including being able to apply for a HC2 certificate to cover prescription costs (Office for Health Improvement and Disparities, 2014). However, individuals report difficulties in preparing application documents (Kang et al., 2019). Uncertainty surrounding financial entitlement to maternity care has led to confounding effects on mental and physical health for mothers (Nellums et al., 2021), especially for undocumented migrants who find it more difficult to access financial support due to legalities (Feldman, 2021). Refugees, asylum seekers, and undocumented migrants cite troubles covering indirect costs incurred from travelling to the destination (Kang et al., 2019; Alkahtani et al., 2014). Difficulties covering costs for breastfeeding formula (Hufton and Raven, 2016), healthy food (Isaacs et al., 2020), and dental care materials (Yuan, 2019) are also reported, suggesting that further support is needed for these populations beyond primary care consultations.

The intangible cost of time is an overlooked barrier pertaining healthcare access. Courses offered for vulnerable populations to understand their entitlements (Jackson-Blott et al., 2015) carry the assumption that refugees, asylum seekers, and undocumented migrants are afforded the privileges of time to reflect on their health and learn a new language, considering that they may be facing difficult circumstances (Isaacs et al., 2020; Piacentini et al., 2019). Temporal costs have also been reported by practitioners who believe that their care is disrupted by confusion surrounding financial eligibility for refugees, asylum seekers, and undocumented migrants (Poduval et al., 2015; Kang et al., 2019; Murphy et al., 2020; Scott et al., 2022), and their need to understand the fine details that shape the unique circumstances and identities of each patient which may shape how they decide on treatment options. In a qualitative study of interviews conducted with 14 health visitors based in London, one practitioner stated, “how much do I invest in sort of really going with their identified needs when there are other families who are new, who are resident, who are entitled to services[…]?” (Burchill and Pevalin, 2014, p.155). There is an ongoing conflict between the intangible aspects of cultural awareness and the tangible aspects of providing care plans, prescriptions, and follow-up appointments, whilst meeting the intense demand. Thus, there is a need for an efficient, holistic system which balances the interests of both.

#### 3.3.5 Acceptability; tangibility of community

**Fig. 6:**
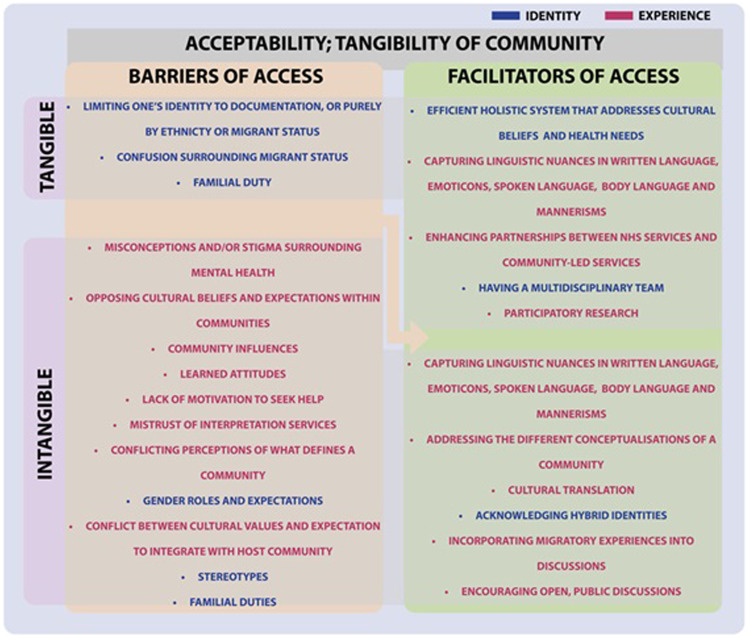
Turning barriers into facilitators of access within the domain of Acceptability; Tangibility of Community.

Acceptability constitutes of the attitudes and views of patients and practitioners (Penchansky and Thomas, 1981). Some communities may significantly influence perceptions of what is deemed acceptable in relation to primary care access (Fig. 6). This section unearths how mental health stigma, gender roles, and expectations deter help-seeking behaviour.

Acceptance of primary care services can be reduced by mental health stigma. Those with mental health issues often do not approach available mental health services (Dura-Vila et al., 2013; Hussain et al., 2021; Scott et al., 2022; Dunn et al., 2021; Paudyal et al., 2021; Strang and Quinn, 2021). Reasons include opposing cultural beliefs which may not facilitate discussions surrounding the awareness and manifestation of mental health issues (Rabiee and Smith, 2013; Mudyarabikwa et al., 2022; Quinn, 2014; Bahu, 2019; Paudyal et al., 2021; Hussain et al., 2021), as well as mistrust of the interpretation services involved (Rayment-Jones et al., 2021b). In an ethnography study of the Somali community, carers, and Somali refugees in the UK with visual impairment (*n* = 88), a refugee demonstrated “learned helplessness”, taught by the community (Higginbottom et al., 2014, p. 197). This may lead to a reduced motivation to access primary care.

Acceptability can be impaired when there is conflict between one’s definition of belonging and the expectations of integrations by the host community. However, when considering intangible expectations within an individual’s community, there are also opposing beliefs within communities, as well as conflicting perceptions of what defines a community. Some believe in the importance of cohesion (Jackson-Blott et al., 2015; Hoare et al., 2020; Paudyal et al., 2021), but others stress that different perspectives and cultural beliefs within a community can cause divides (Quinn, 2014; Kaneoka and Spence, 2020; Paudyal et al., 2021; Hussain et al., 2021). For instance, people who migrated for economic reasons may not entirely understand the challenges faced by those who migrated forcibly even if they share a similar culture. Therefore, assessing an individual’s place in society according to the relationship with their community, as illustrated in Berry’s (1997) model, is not universally acceptable as different people have various conceptualisations of what a community is, who belongs in it, and the significance of a community to their way of life.

The role of gender-based expectations in shaping acceptability of healthcare services within communities is not well understood. Women face various reported issues such as poorer general health compared to men (Cheung and Phillimore, 2017), complications of female genital mutilation (Burchill and Pevalin, 2014), poorer prenatal outcomes compared to citizens (Goodwin et al., 2021; Rayment-Jones et al., 2021a), difficulties breastfeeding due to physical and mental stress (Hufton and Raven, 2016), domestic violence (Feldman, 2021), and political gender-based violence (Palattiyil and Sidhva, 2021). Differences in gender-based expectations within one’s culture and the expectation to integrate in society has led to mental deterioration (Burchill and Pevalin, 2014), especially when in conflict (Morrice, 2017; Kaneoka and Spence, 2020; Paudyal et al., 2021). This challenges the positive association with integration. It also sheds light on women being the subaltern when they are unable to discuss issues relating to their health due to gender-based expectations, and this could affect help-seeking behaviour. Holding public participatory workshops within the community can open discussions between community members (Quinn, 2014; Strang and Quinn, 2021), and give the subaltern a direct means of communicating, whilst spreading awareness of mental health in the community. This could help to improve acceptability from the viewpoint of the patients, the wider community, and practitioners.

Language and emotional processing are tied with cultural influences, which can alter how acceptability of primary care within communities is understood by researchers. Questionnaires have been used as a methodology to capture the views of refugees, asylum seekers, and undocumented migrants. They provide the benefit of anonymity to encourage participation (Morgan et al., 2017), and enable the application of quantitative methods to support qualitative research (Murphy et al., 2020; Rayment-Jones et al., 2021a; Samkange-Zeeb et al., 2020). Emoticons are used to capture satisfaction of these populations without the need for English (Scott et al., 2022). However, the interpretation of certain emoticons may be affected by cultural subjectivity. In a quantitative and qualitative evaluation of 17 first-generation Tamil participants living in the UK, it was noted that they found it “hard […] to put a number to their feelings” (Bahu, 2019, p. 17), illustrating how numerically reconstructing experiences may be difficult for those who are unfamiliar with this. Furthermore, questionnaires often require translations (Dura-Vila et al., 2013; Falla et al., 2017; Morgan et al., 2017), which may lose potential nuances within a language. Interview transcripts may include body language notation, but interpretation can be influenced by cultural experiences. Whilst a study has tried to overcome this by recruiting translators external to the study (Higginbottom et al., 2014), it would have been beneficial to have these transcripts reviewed by an anthropologist who is familiar with cultural practices.

Limited knowledge of identities can influence assumptions made by practitioners about what is deemed acceptable for a certain patient demographic. Practitioners recognise the linguistic and cultural barriers of accessing mental health support (Burchill and Pevalin, 2014; Dunn et al., 2021). However, confusion surrounding identities have been reported by practitioners (Papageorgiou et al., 2020). Categories such as ethnicity and migrant status are often needed to facilitate healthcare systems (Hargreaves et al., 2020). Inadequate approaches to their medical care may arise when stereotyping patients according to their ethnic background, rather than looking more deeply at reasons for migrating and one’s migratory experiences (Rabiee and Smith, 2013; Hussain et al., 2021; Gazard et al., 2015). This highlights the importance of having multidisciplinary teams that can lend different perspectives into a patient’s circumstance. However, the tendency to attribute medical problems to migrant status has proven uncomfortable for some patients (Piacentini et al., 2019). One participant of a qualitative study of HIV-positive asylum seekers in Scotland said, “I have so many problems, I ask myself so many questions…It is just because I am black? Is it just because I am a woman? Is it just because I am HIV? Is it just because I am an asylum seeker?” (Palattiyil and Sidhva, 2021, p. 272), illustrating the tension between these intersectional identities. This ties in with Bhaba’s (1994) theory of hybridity, which calls for a need to recognise that people can carry multiple changing identities; as a citizen of their home country and as an individual who is constantly learning about new mannerisms and characteristics of their host community, whilst also possibly facing pressures from legal standpoints and potential duties as a family member. Berry (1997) separates the cultural values of the host community and the community from which refugees, asylum seekers, and undocumented migrants have travelled from, yet it is possible for individuals to harbour identities that is an amalgamation of both values. This signifies the importance of building connections between NHS services and community-led services to foster educational dialogue and improve the acceptability of hybrid identities.

#### 3.3.6 Awareness; tangibility of the past and present

**Fig. 7:**
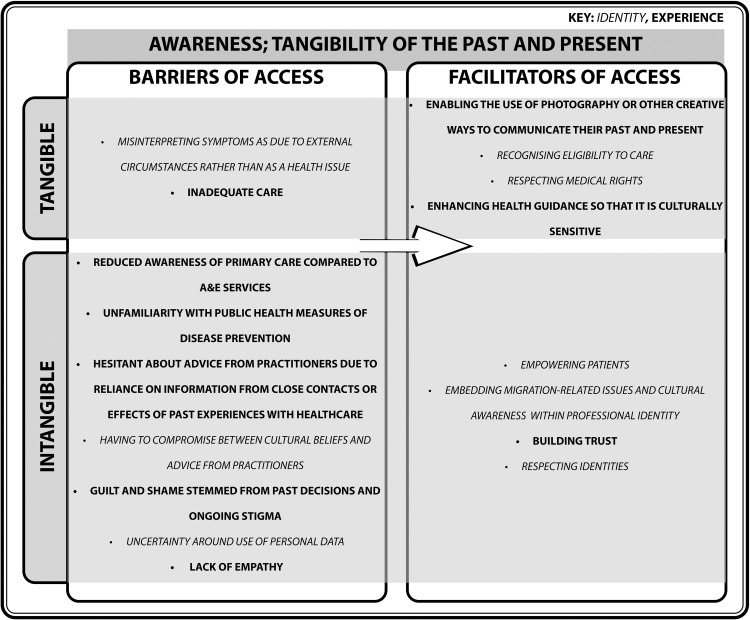
Turning barriers into facilitators of access within the domain of Awareness; Tangibility of the Past and Present.

Saurman’s (2016) addition of the Awareness domain to Penchansky and Thomas’ theory reflects on the current knowledge of individuals. Past interactions and ongoing power dynamics can influence assumptions of primary care, and how this shapes the uptake of health-related advice (Fig. 7).

Awareness of primary care services are influenced by preconceptions of healthcare infrastructure and sustained dependency on community ties. There is a gap in awareness about the services available among refugees, asylum seekers, and undocumented migrants who particularly come from countries that do not largely enforce public health measures or have different healthcare systems (Kang et al., 2019). Undocumented migrants demonstrate a greater awareness of their rights to free emergency services compared to their rights to access primary care (Poduval et al., 2015). There is hesitancy to act on advice surrounding healthy eating (Burchill and Pevalin, 2014), parental behaviour (Burchill and Pevalin, 2014), screening (Higginbottom et al., 2014; Falla et al., 2017), sex education (Kaneoka and Spence, 2020), vaccination uptake (Perry et al., 2020), and prevention of oral diseases (Sarri et al., 2012). Instead, it is common for health-related knowledge and advice to be passed within families and communities (Burchill and Pevalin, 2014; Morrice, 2017; Strang and Quinn, 2021), thus, may explain why some migrants are drawn to communities that share similar backgrounds. For example, some communities stress the importance of breastfeeding in cultural and religious contexts (Hufton and Raven, 2016). This is perceived as ‘separation’ in Berry’s (1997) model, but this decision may be most beneficial to boost awareness of health-related matters in circumstances where information from public health bodies may not reach them.

On the other hand, awareness may be compromised when cultural practices are in conflict with advice from primary care. For example, some patients are advised not to breastfeed due to medical reasons, such as being HIV-positive (Hufton and Raven, 2016; Palattiyil and Sidhva, 2021), despite breastfeeding being of significance to some cultures, whilst also considering that some conditions, such as being HIV-positive, is stigmatised in some cultures (Whyte et al., 2015). Feelings of guilt and shame have been reported due to leaving one’s home country behind, as well as not being welcomed by the host community (Bahu, 2019; Hoare et al., 2020). Berry’s (1997) model implies that marginalisation is an individual’s choice, but communities themselves can cause one to become marginalised. Furthermore, the complex socioeconomic circumstances faced by refugees, asylum seekers, and undocumented migrants have led to some participants attributing symptoms to their physical situation rather than serious infections such as tuberculosis (Craig et al., 2014). This illustrates a difficulty for one to interpret when their identity as a patient should emerge, highlighting the need for increased awareness of how to recognise eligibility for primary care access whilst respecting cultural values.

Awareness of services may also be hindered for those who are unable to directly communicate with services due to linguistic barriers, and are therefore considered the subaltern in line with Spivak’s (1995) theory. Encouraging other means of communication may boost awareness, and improve engagement with practitioners (Morrice, 2017). For example, a study by Ortega-Alcázar and Dyck (2012) shows participants taking photos of aspects of their community which are conducive to their wellbeing. In this way, participants are directly contributing to the definition of terms such as ‘health’, thus, is a powerful tool to communicate with non-English speakers about their past experiences and present needs. This can be coupled with the tangibility of social cues, including the consideration of body language, photography, and conversational nuances, to improve outcomes of consultations.

Forced migrants are subject to difficult circumstances which illustrate that awareness of these services is futile without reciprocation from practitioners. For instance, the NHS is jointly responsible for the care of detainees in immigration detention centres (Home Office Immigration Enforcement, NHS England, and Public Health England, 2018; Hollis, 2019). Despite trust and empathy being crucial aspects of consultations (Yuan, 2019; Papageorgiou et al., 2020), there are reports of inadequate care and a lack of empathy in healthcare services operating within these centres (Hollis, 2019; Palattiyil and Sidhva, 2021). Despite these patients being aware of the services they had access to, they were not granted the care that they needed.

Reviews of GP-patient interactions demonstrate persistent asymmetries of power (Pilnick and Dingwall, 2011; Ziebland et al., 2021; Advocat et al., 2025), particularly so for migrants. There is uncertainty among patients and practitioners about how data may be shared with government or immigration authorities, which has discouraged some from registering with primary care (Murphy et al., 2020; Mudyarabikwa et al., 2022; Nellums et al., 2021). Practitioners have emphasised the importance of integrating migration-related issues into their professional identity, rather than treating them as peripheral concerns (Apostolidou, 2015). This highlights the need for heightened awareness of the cultural, digital, and political factors that shape how refugees, asylum seekers, and undocumented migrants navigate healthcare. Past experiences of exclusion and surveillance may also influence how comfortable they feel discussing certain aspects of their health. In a qualitative study of semi-structured interviews conducted with 8 specialist professionals, Apostolidou argues that such experiences have encouraged some professionals to incorporate within their identity the “knowledge, expertise, and power […] to combat the social injustices and to benefit politically and socially disadvantaged groups like refugees” (Apostolidou, 2015, p.501). This dynamic illustrates the intangible forms of power operating within practitioner–patient relationships, where clinicians are positioned as potential agents of social change without direct understanding of their patients’ lived realities. Empowering refugees, asylum seekers, and undocumented migrants to develop a stronger sense of agency within their own identities may therefore be crucial in reducing hesitancy to engage with primary care.

## 4 Discussion – conclusions

We conducted a critical interpretive synthesis of 49 papers, which met the inclusion criteria, concerning barriers and facilitators of primary care access for refugees, asylum seekers, and undocumented migrants in the UK. To our knowledge, this is the first review to address the issue of access to primary care through a post-colonial theoretical viewpoint. We developed the *Tangibility of Access* model through the synthesis, which outlines six domains of experience and identity that affect primary care access. Tangible barriers to access, such as financial costs and translated material, may be addressed by providing the necessary resources and monetary support. However, intangible aspects such as cultural translation and hybrid identities have received less research attention.

As demonstrated in the *Tangibility of Access* model, the identities and experiences of refugees, asylum seekers, and undocumented migrants have been conceptualised as tangible and intangible barriers and facilitators of primary care access, in the form of social cues, location, networks, expense, community and the past and present. Persistent barriers demonstrates that monodisciplinary research does not address barriers in its entirety. Healthcare systems remain driven by quantitative performance metrics that may overlook experiential dimensions of access (De Melo Santos et al., 2025), even though many intangible barriers exist (Fig. 2, Fig. 3, Fig. 4, Fig. 5, Fig. 6, Fig. 7). By framing them in the *Tangibility of Access* model, it gives intangible barriers a visible platform. Additionally, a focus on the interplay between identity and experience could reveal how structural and cultural forces jointly constrain access, thereby informing interventions that address both procedural and relational barriers .

Using an interdisciplinary perspective, we were able to take a different lens on Penchansky and Thomas’ (1981) healthcare-based theory by including post-colonial insights, such as hybrid identities, unspoken perspectives, and expectations of host and migrant communities. In turn, we generated the *Tangibility of Access* model. This has illustrated that the assessment of primary care access can be improved by increasing holistic investigations into the tangible and intangible issues concerning health, help-seeking behaviour, and consultations, such as the role of empathy in consultations, and how the impact of cultural attitudes concerning sexuality and health can affect perceptions surrounding eligibility to care. This responds to our first research question by demonstrating that access cannot be reduced to service availability or affordability but is co-constructed through relational, cultural, and symbolic processes.

This model further identified a lack of research surrounding intangible identities which shape the perception of accessing primary care. This addresses our second research question about how these identities and experiences influence access, and highlights the importance of recognising emotional and symbolic forms of exclusion that persist even when formal barriers are removed. Addressing these requires both reflexive practice among clinicians and co-designed community engagement to rebuild trust. To confront challenges surrounding continuity of care (Harris et al., 2022), there is a greater need to challenge assumptions about patient identities, open more spaces for discussion, and proactively respond to potential antagonism between available primary care services and cultural values. Confusion surrounding migrant status (Masocha and Simpson, 2012), could be reduced by improving data collection (Gazard et al., 2015; Kearns et al., 2017; Nellums et al., 2021). However, providing cultural and migrant-related education, as well as encompassing intangible identities is essential to reduce stereotypes and assumptions derived from categorisation, and ultimately improve primary care access. Persistent linguistic barriers (Bhatia and Wallace, 2007; Bellamy et al., 2015; Jallow et al., 2021; Tankwanchi et al., 2021) suggests that it is not sufficient to increase translation services, but to also ensure that they are culturally informed.

Our third research question considered implications for policy and practice; the findings underscore the need for integrated strategies that bring together practitioners, policymakers, and community representatives to translate intangible facilitators such as trust, belonging, and perceived legitimacy into tangible service improvements. Examples include embedding trauma-informed and rights-based principles in GP registration policies and expanding Safe Surgery-type initiatives nationwide. By using an interdisciplinary methodological approach, a theme of vulnerability emerged. Emphasising vulnerability rather than empowerment may reduce the likelihood of these populations accessing healthcare (Bradby et al., 2019).

Future research should therefore evaluate how empowerment-oriented interventions (co-designed with refugees, asylum seekers, and undocumented migrants) can strengthen agency and mitigate structural vulnerability. It would be useful to learn directly from refugees, asylum seekers, and undocumented migrants about how they would like their identities to be understood, rather than assuming that it is dictated by their migrant status or ethnicity. Due to increasing pressure for emergency services in the UK (Graetz et al., 2017; Phung et al., 2020; Aspinall, 2014), a deeper insight into how tangible and intangible identities and experiences can be turned into facilitators, with the feedback of multiple stakeholders, including practitioners, policymakers, and community members, is necessary to guide patients to the relevant primary care services, and access preventative measures that are best for them in the given circumstances. Developing participatory models of service design, informed by intersectional and structural vulnerability frameworks, would not only refine the Tangibility of Access model but also ensure that policy reforms address both the material and relational dimensions of access.

### 4.1 Limitations

This review has several limitations. The search was restricted to English-language publications and to the past decade, which may omit earlier work. Some studies lacked detailed reporting of migrant status, limiting comparability. The exclusion of papers that did not explicitly mention immigrant status in titles or abstracts may have omitted studies including migrants not identified as such. Conceptually, the synthesis depends on our interpretive framework, and data heterogeneity constrained meta-analytic assessment. Due to time constraints, grey literature was not included in this research. Consultation of these resources would highlight further assumptions that surround the identities and experiences of refugees, asylum seekers and undocumented migrants. However, it is likely that reports which rely on primary data follow similar assumptions and constructions which have so far been identified.

A limitation of the critical interpretive approach is that it is not as reproducible compared to other stringent systematic reviews (Dixon-Woods et al., 2006). Additionally, the definition of access can vary throughout the literature and thus required a definition. To overcome these two issues, Penchansky and Thomas’ (1981) theory was used to guide the results of the paper to provide a level of reproducibility. Although, the framework itself was subject to interpretation and critique throughout the discussion to generate theory.

### 4.2 Recommendations for further research, policy and practice

Further interdisciplinary research is necessary to uncover how certain cultural nuances within behaviour and speech can affect vulnerable individuals, in tangent with their migratory experiences. This may induce a shift of policy approaches away from one that is predominantly guided by tangible measures, defined by documentation and medical services, and towards approaches that incorporate the intangible influences of mental conceptualisations and cultural values which affect healthcare access and consultations. Examples include the Safe Surgeries initiative (Davidson et al., 2023), which trains primary-care teams to register patients regardless of documentation status, and Health Access Cards used in Newcastle that summarise entitlements and multilingual support. Incorporating such trauma-informed, community-led approaches may enhance the applicability of our findings.

In conclusion, a critical interpretive synthesis of published research relating to access to primary healthcare in the UK for refugees, asylum seekers, and undocumented migrants identified a new synthetic concept of *Tangibility of Access*. The constructed identities and experiences of refugees, asylum seekers and undocumented migrants were analysed using Penchansky and Thomas’ (1981) theory of healthcare access, with the additional input from Saurman (2016), and three post-colonial theories, drawing on the works of Bhaba (1994), Spivak (1995), and Berry (1997). Post-colonial theory was instrumental in challenging given assumptions within the literature concerning the positive association with integration, the hybridity of identities, and the recognition of the subaltern. The generation of the *Tangibility of Access* model has shed light on the need for more research on intangible barriers which may reveal why refugees, asylum seekers, and undocumented migrants are possibly continuing to face difficulties in accessing primary care in circumstances where the resources and services are physically available for use. Barriers and facilitators of primary care access may also be better understood by looking at the interactions between the identities and experiences of these populations. Ultimately, this research illustrates the need for further interdisciplinary inquiries into intangible identities and experiences, the assumptions underlying tangible identities and experiences, and the ways in which refugees, asylum seekers, and undocumented migrants can be empowered to address their needs within the current UK primary care system.

## Funding sources

This research did not receive any specific grant from funding agencies in the public, commercial, or not-for-profit sectors. Georgia Black acknowledges funding from THIS Institute, University of Cambridge (RG88620/PD-2019-02-004), and Barts Charity (G-001520).

## CRediT authorship contribution statement

**Jeniffer Jeyason:** Conceptualization, Formal analysis, Methodology, Writing – original draft, Writing – review & editing. **Georgia B. Black:** Conceptualization, Supervision, Writing – original draft, Writing – review & editing.

## Declaration of competing interest

The authors declare that they have no known competing financial interests or personal relationships that could have appeared to influence the work reported in this paper.

## Figures and Tables

**Table:** 

#	Query
1	Community Health
2	health care
3	primary care
4	health worker
5	general practice.mp.
6	primary care.mp
7	family physician.mp.
8	"Family Practitioner*"/
9	general practitioner.mp.
10	help seeking.mp.
11	caring behaviour.mp.
12	family medicine.mp.
AND	
13	refugee/
14	asylum seeker/ or immigrant/
15	migrant worker/
16	immigrant population/
17	forced migration/
18	migrants experience/
19	migration determinant/
20	(displaced person or displaced people).mp. [mp=title, abstract, geobase descriptor, heading word, original title, keyword]
21	expatriate.mp.
22	undocumented migrant.mp.

**Table:** 

#	Query
1	community health services/ or community health nursing/ or community pharmacy services/
2	general practice/ or family practice/
3	primary health care/ or "continuity of patient care"/ or patient-centered care/
4	physicians, family/ or physicians, primary care/
5	"delivery of health care"/ or culturally competent care/ or
	"delivery of health care, integrated"/ or health services accessibility/ or healthcare disparities/ or practice patterns, pharmacists'/ or practice patterns, dentists'/ or
	practice patterns, nurses'/ or practice patterns, physicians'/
6	General Practitioners/
7	"Patient Acceptance of Health Care"/
8	"Attitude of Health Personnel"/
9	primary care.mp.
10	family practitioner.mp.
11	help seeking.mp.
12	caring behaviour.mp.
13	family medicine.mp.
14	(primary adj2 care).tw.
AND	
15	Refugees/
16	"Transients and Migrants"/
17	asylum seek*.mp.
18	migrant*.mp.
19	("displaced person*" or "displaced people").mp. [mp=title, abstract, original title, name of substance word, subject heading word, floating sub-heading word, keyword heading word, organism supplementary concept word, protocol supplementary concept word, rare disease supplementary concept word, unique identifier, synonyms
20	(immigration or migration).mp. [mp=title, abstract, original title, name of substance word,
	subject heading word, floating sub-heading word, keyword heading word, organism
	supplementary concept word, protocol supplementary concept word, rare disease
	supplementary concept word, unique identifier, synonyms]
21	expatriate*.mp.
22	("second generation" or "second-generation" or "2nd generation").mp.
	[mp=title, abstract, original title, name of substance word, subject heading word,
	floating sub-heading word, keyword heading word, organism supplementary concept word,
	protocol supplementary concept word, rare disease supplementary concept word,
	unique identifier, synonyms]
23	("first generation" or "first-generation" or "1st generation").mp.
	[mp=title, abstract, original title, name of substance word, subject heading word,
	floating sub-heading word, keyword heading word, organism supplementary concept word,
	protocol supplementary concept word, rare disease supplementary concept word,
	unique identifier, synonyms]
24	"undocumented migrant*".mp.
25	"forced migrant".mp.

**Table:** 

#	Query
1	community health/ or health service needs/
2	pharmacy/ or pharmacists/
3	general practitioners/ or family medicine/ or family physicians/
4	primary health care/
5	health care delivery/ or health care access/ or
	health care utilization/ or "quality of care"/ or
	"quality of services"/ or health disparities/ or
	health service needs/ or patient centered care/ or
	treatment barriers/
6	help seeking behavior/ or health care seeking behavior/
7	caring behaviors/
8	general practice.mp.
9	"family practitioner*".mp.
10	(primary adj2 care).tw.
AND	
11	refugees/ or asylum seeking/ or immigration/ or political asylum/
12	immigration/ or expatriates/
13	migrant farm workers/ or migrant workers/
14	("displaced person*" or "displaced people").mp. [mp=title, abstract, heading word, table of contents, key concepts, original title, tests & measures, mesh word]
15	("second generation" or "second-generation" or "2nd generation").mp.
	[mp=title, abstract, heading word, table of contents, key concepts, original title, tests & measures, mesh word]
16	("first generation" or "first-generation" or "1st generation").mp.
	[mp=title, abstract, heading word, table of contents, key concepts,
	original title, tests & measures, mesh word]
17	undocumented migrant*
18	"forced migrant".mp.

**Table:** 

#	Query
1	((((((((((((((ALL=(community health))) OR ALL=(general practice)))) OR ALL=(Primary Care)) OR ALL=(primary care)) OR ALL=(primary health care)) OR ALL=(Family physician)) OR ALL=(Family Practitioner)) OR ALL=(General Practitioner)) OR ALL=(help seeking)) OR ALL=(help-seeking)) OR ALL=(caring behavi*)) OR ALL=(Family medicine)
AND	
2	(((((((((((((((ALL=(asylum seek*))) OR ALL=(refugee*)) OR ALL=(immigrant*)) OR ALL=(displaced person)) OR ALL=(displaced people)) OR ALL=(immigration)) OR ALL=(expatriate*)) OR ALL=(undocumented migrant*)) OR ALL=(forced migra*)))))))
AND	
3	ALL=(UNITED KINGDOM OR UK OR U.K. OR ENGLAND OR WALES OR SCOTLAND OR NORTHERN IRELAND OR BRITAIN)

**Table:** 

#	Query
1	MAINSUBJECT.EXACT("Helpseeking") OR MAINSUBJECT.EXACT("Community health care") OR MAINSUBJECT.EXACT("General practitioner hospitals") OR MAINSUBJECT.EXACT("Community health") OR MAINSUBJECT.EXACT("Primary care trusts") OR MAINSUBJECT.EXACT("General practitioners") OR MAINSUBJECT.EXACT("General practice")
AND	
2	MAINSUBJECT.EXACT("Statelessness") OR MAINSUB
	JECT.EXACT("Asylum") OR MAINSUBJECT.EXACT("Refugees") OR MAINSUBJECT.EXACT("Displaced people") OR MAINSUBJECT.EXACT("Immigration")

**Table:** 

#	Query
1	MAINSUBJECT.EXACT("Community health care") OR MAINSUBJECT.EXACT("Health services utilization") OR MAINSUBJECT.EXACT("Primary care") OR MAINSUBJECT.EXACT("Family physicians") OR "community health" OR "general practice" OR "primary care" OR "primary health care" OR "Family Practitioner*" OR "General Practitioner*" OR "help seeking" OR "help-seeking" OR "Caring Behavi*" OR "Family medicine"
AND	
2	MAINSUBJECT.EXACT("Immigration") OR MAINSUBJECT.EXACT("Statelessness") OR MAINSUBJECT.EXACT("Displaced persons") OR MAINSUBJECT.EXACT("Political asylum") OR MAINSUBJECT.EXACT("Refugees") OR "second generation" OR "second-generation" OR "2nd generation" OR "first-generation" OR "first generation" OR "1st generation" OR "displaced people" OR "migration" "expatriate*" OR "undocumented migrant*" OR "forced migra*"

**Table:** 

Citation (Author(s), Year and Title)	Quality Assessment				
	Are the aims/objectives of the study explicitly stated?	Is the methods section in line with the intended research question(s)?	Is the methodology well-explained?	Are the results described clearly?	Does the analysis approach follow a clear, logical progression?
Alkahtani et al., 2014. Access to medicines by child refugees in the East Midlands region of England: a cross-sectional study [Article].	✓	✓	✓	✓	✓
Apostolidou, 2015. Politicised notions of professional identity and psychosocial practice among practitioners working with asylum seekers and refugees [Empirical Study; Interview; Qualitative Study].		✓		✓	✓
Armitage et al. 2021. Description and evaluation of a pathway for unaccompanied asylum-seeking children [Article; Early Access].	✓	✓	✓	✓	✓
Bahu, 2019. War, trauma and culture: Working with Tamil refugees and asylum seekers using culturally adapted CBT [Empirical Study; Quantitative Study].	✓	✓	✓	✓	✓
Burchill and Pevalin, 2014. Demonstrating cultural competence within health-visiting practice: working with refugee and asylum-seeking families.		✓	✓	✓	✓
Cheung and Phillimore, 2017. Gender and Refugee Integration: a Quantitative Analysis of Integration and Social Policy Outcomes.		✓	✓	✓	✓
Craig et al., 2014. 'Complex' but coping: experience of symptoms of tuberculosis and health care seeking behaviours - a qualitative interview study of urban risk groups, London, UK [Article].	✓	✓	✓	✓	✓
Dunn et al., 2021. Service evaluation of the english refugee health information system: Considerations and recommendations for effective resettlement.	✓	✓	✓	✓	
Dura-Vila et al., 2013. Mental health problems of young refugees: Duration of settlement, risk factors and community-based interventions [Article].	✓	✓	✓	✓	✓
Falla et al., 2017. Language support for linguistic minority chronic hepatitis B/C patients: an exploratory study of availability and clinicians' perceptions of language barriers in six European countries [Article].		✓	✓	✓	✓
Feldman, 2021. NHS charging for maternity care in England: Its impact on migrant women.		✓	✓	✓	✓
Gazard et al., 2015. Challenges in researching migration status, health and health service use: an intersectional analysis of a South London community.	✓	✓	✓	✓	
Goodwin et al., 2021. Addressing social inequity through improving relational care: A social-ecological model based on the experiences of migrant women and midwives in South Wales [Article; Early Access].	✓	✓	✓	✓	✓
Hargreaves et al., 2020. Delivering multi-disease screening to migrants for latent TB and blood-borne viruses in an emergency department setting: A feasibility study [Article].	✓	✓	✓	✓	
Higginbottom et al., 2014. Health and Social Care Needs of Somali Refugees With Visual Impairment (VIP) Living in the United Kingdom: A Focused Ethnography With Somali People With VIP, Their Caregivers, Service Providers, and Members of the Horn of Africa Blind Society [Article].	✓	✓	✓	✓	✓
Hoare et al., 2020. How do people seeking asylum in the United Kingdom conceptualize and cope with the asylum journey?	✓	✓	✓	✓	✓
Hollis, 2019. The psychosocial experience of UK immigration detention [Article].	✓	✓	✓		✓
Hufton and Raven, 2016. Exploring the infant feeding practices of immigrant women in the North West of England: a case study of asylum seekers and refugees in Liverpool and Manchester [Article].	✓	✓	✓	✓	✓
Hussain et al., 2021. Recognizing service users’ diversity: social identity narratives of British Pakistanis in a mental health context.	✓	✓	✓		
Isaacs et al., 2020. 'I don't think there's anything I can do which can keep me healthy': how the UK immigration and asylum system shapes the health & wellbeing of refugees and asylum seekers in Scotland [Article; Early Access].		✓	✓	✓	✓
Jackson-Blott et al., 2015. Evaluating a "healthy minds" course for asylum seekers [Article].	✓	✓		✓	✓
Kaneoka and Spence, 2020. The cultural context of sexual and reproductive health support: an exploration of sexual and reproductive health literacy among female Asylum Seekers and Refugees in Glasgow [Sexual and reproductive health support].	✓	✓	✓	✓	
Kang et al., 2019. Access to primary health care for asylum seekers and refugees: a qualitative study of service user experiences in the UK [Article].	✓	✓	✓	✓	✓
Kearns et al., 2017. Healthy Migrants in an Unhealthy City? The Effects of Time on the Health of Migrants Living in Deprived Areas of Glasgow.	✓	✓	✓	✓	
MacVicar et al., 2015. What are the CPD needs of GPs working in areas of high deprivation? Report of a focus group meeting of 'GPs at the Deep End' [Article].	✓	✓	✓	✓	✓
Morgan et al., 2017. Exploring the relationship between postmigratory stressors and mental health for asylum seekers and refused asylum seekers in the UK [Empirical Study; Quantitative Study].	✓	✓	✓	✓	
Morrice, 2017. Cultural values, moral sentiments and the fashioning of gendered migrant identities [Article].		✓	✓		✓
Mudyarabikwa et al., 2022. Refugee and Immigrant Community Health Champions: a Qualitative Study of Perceived Barriers to Service Access and Utilisation of the National Health Service (NHS) in the West Midlands, UK [Article].	✓	✓	✓	✓	✓
Murphy et al., 2020. Healthcare access for children and families on the move and migrants [Article].	✓	✓	✓	✓	✓
Negron, 2018. Supporting asylum seekers and refugees who suffer chronic pain: an experience [Article].	✓	✓	✓	✓	✓
Nellums et al., 2021. "It's a life you're playing with": A qualitative study on experiences of NHS maternity services among undocumented migrant women in England [Empirical Study; Interview; Qualitative Study].	✓	✓	✓	✓	✓
Ortega-Alcázar and Dyck, 2012. Migrant narratives of health and well-being: Challenging 'othering' processes through photo-elicitation interviews.		✓	✓	✓	
Palattiyil and Sidhva, 2021. HIV, Human Rights and Migration: Narratives of HIV-Positive Asylum Seekers in Scotland [Article].	✓	✓	✓		
Papageorgiou et al., 2020. Patient data-sharing for immigration enforcement: a qualitative study of healthcare providers in England [Article].	✓	✓	✓	✓	✓
Paudyal et al., 2021. Qualitative study on mental health and well-being of Syrian refugees and their coping mechanisms towards integration in the UK [Article].	✓	✓	✓	✓	✓
Perry et al., 2020. Inequalities in vaccination coverage and differences in follow-up procedures for asylum-seeking children arriving in Wales, UK [Article].	✓	✓	✓		✓
Piacentini et al., 2019. Moving beyond the 'language problem': developing an understanding of the intersections of health, language and immigration status in interpreter-mediated health encounters [Article].	✓	✓	✓	✓	✓
Poduval et al., 2015. Experiences among undocumented migrants accessing primary care in the United Kingdom: a qualitative study.	✓	✓	✓	✓	✓
Priebe et al., 2013. Mental health-care provision for marginalized groups across Europe: findings from the PROMO study [Article].	✓	✓	✓	✓	✓
Quinn, 2014. Participatory action research with asylum seekers and refugees experiencing stigma and discrimination: the experience from Scotland [Article].		✓	✓	✓	✓
Rabiee and Smith, 2013. Being understood, being respected: An evaluation of mental health service provision from service providers and users' perspectives in Birmingham, UK [Empirical Study; Interview; Focus Group; Qualitative Study].	✓	✓	✓	✓	✓
Rayment-Jones, Dalrymple, et al., 2021a. Project20: Does continuity of care and community-based antenatal care improve maternal and neonatal birth outcomes for women with social risk factors? A prospective, observational study [Article].	✓	✓	✓	✓	✓
Rayment-Jones, Harris, et al., 2021b Project20: interpreter services for pregnant women with social risk factors in England: what works, for whom, in what circumstances, and how?	✓	✓	✓	✓	
Samkange-Zeeb et al., 2020. Superdiversity, migration and use of internet-based health information - results of a cross-sectional survey conducted in 4 European countries [Article].	✓	✓	✓	✓	✓
Sarri et al., 2012. A school-based epidemiological study of dental neglect among adolescents in a deprived area of the UK [Article].	✓	✓	✓	✓	✓
Scott et al., 2022. Refugee, Migrant and Asylum Seekers' Experience of Accessing and Receiving Primary care in a UK City of Sanctuary [Article].	✓	✓	✓	✓	✓
Strang and Quinn, 2021. Integration or Isolation? Refugees' Social Connections and Wellbeing [Article].	✓	✓	✓	✓	✓
Whyte et al., 2015. A study of HIV positive undocumented African migrants' access to health services in the UK [Empirical Study; Interview; Qualitative Study].	✓			✓	✓
Yuan, 2019. Evaluating an Oral Health Education Intervention in Chinese Undocumented Migrant Mothers of Infants in Northern Ireland [Article].	✓	✓	✓	✓	✓

## APPENDIX A – SEARCH TERMS

**Database: GEOBASE**

**Database: Ovid MEDLINE**

**Database: PSYCINFO**

**Database: Web of Science**

**Database: Applied Social Sciences Index & Abstracts (ASSIA)**

**Database: International Bibliography of the Social Sciences (IBSS)**
